# Self-injury and aggression in tuberous sclerosis complex: cross syndrome comparison and associated risk markers

**DOI:** 10.1186/1866-1955-6-10

**Published:** 2014-05-10

**Authors:** Kate E Eden, Petrus J de Vries, Jo Moss, Caroline Richards, Chris Oliver

**Affiliations:** 1 Cerebra Centre for Neurodevelopmental Disorders, School of Psychology, University of Birmingham, Birmingham B15 2TT, UK; 2 Division of Child & Adolescent Psychiatry, University of Cape Town, 46 Sawkins Road, Rondebosch, Cape Town 7700, South Africa; 3 Institute of Cognitive Neuroscience, University College London, Alexandra House, 17-19 Queen Square, London WC1N 3AR, UK

**Keywords:** Aggression, ASD, Impulsivity, Pain, Repetitive/stereotyped behaviour, Self-injury, Tuberous sclerosis complex

## Abstract

**Background:**

Research reporting prevalence rates of self-injurious and aggressive behaviour in people with tuberous sclerosis complex (TSC) is limited. No studies have compared rates of these behaviours in TSC with those in other syndrome groups matched for degree of disability or investigated risk markers for these behaviours in TSC.

**Methods:**

Data from the Challenging Behaviour Questionnaire were collected for 37 children, aged 4 to 15 years, with TSC. Odds ratios were used to compare rates of self-injury and aggression in children with TSC with children with idiopathic autism spectrum disorder (ASD), fragile X, Cornelia de Lange and Down syndromes. Characteristics were measured using the Mood Interest and Pleasure Questionnaire, the Activity Questionnaire, the Social Communication Questionnaire, the Repetitive Behaviour Questionnaire, the Wessex Behaviour Schedule and the revised Non-communicating Children Pain Checklist. Mann-Whitney *U* analyses were used to compare characteristics between individuals with self-injury and aggression and those not showing these behaviours.

**Results:**

Rates of self-injury and aggression in TSC were 27% and 50%, respectively. These are high but not significantly different from rates in children with Down syndrome or other syndrome groups. Both self-injury and aggression were associated with stereotyped and pain-related behaviours, low mood, hyperactivity, impulsivity and repetitive use of language. Children who engaged in self-injury also had lower levels of interest and pleasure and showed a greater degree of ‘insistence on sameness’ than children who did not self-injure. Aggression was associated with repetitive behaviour. The majority of these associations remained significant when the association with level of adaptive functioning was controlled for.

**Conclusions:**

Behavioural profiles can be used to identify those most at risk of developing self-injury and aggression. Further research is warranted to understand the influence of such internal factors as mood, ASD symptomatology and pain on challenging behaviour in people with intellectual disability.

## Background

Tuberous sclerosis complex (TSC) is a genetic disorder resulting from a mutation of either the *TSC1* gene on chromosome 9q34 [1] or the *TSC2* gene on chromosome 16p13 [2]. Estimated prevalence rates range from 1/6,800 to 1/11,400 [3,4] and birth incidence estimates range from 1/6,000 to 1/15,000 [5]. Intellectual ability forms a bimodal distribution, with ≈ 30% of individuals showing profound intellectual disability (ID) and 70% of individuals falling within a normal distribution of intellectual ability, with a mean IQ of 93 [6]. Best estimates from epidemiological populations suggest that about 45% of individuals with TSC have ID [7].

High rates of aggression (13.3% to 58%) [8-10] and self-injury (10% to 41%) [8,10,11] have been reported in people with TSC. However, to date, the rates of self-injurious and aggressive behaviour have not been compared with those reported in other syndrome groups and individuals with ID. Therefore, it is currently unknown whether individuals with TSC are at greater risk of engaging in these behaviours compared with other populations with similar levels of ID.

Several physical and behavioural characteristics associated with TSC indicate that individuals with TSC may be a high-risk group for these behaviours. Firstly, individuals with severe or profound ID are significantly more likely to engage in self-injury and aggression than individuals with mild or moderate ID [12]. Given that 30% of individuals with TSC have profound ID, this elevates the risk for these behaviours. Secondly, TSC is characterized by abnormal growths in multiple organs, which are likely to cause pain and discomfort [13-15]. For example, individuals with TSC may experience subependymal giant cell astrocytoma brain tumours, leading to raised intracranial pressure, headaches and photophobia [16]. They may also experience renal angiomyolipoma, which causes flank pain, bleeding and renal failure [17]. Pain, and behavioural indicators of pain, have been shown to be associated with a higher frequency of self-injurious and aggressive behaviour in people with ID [18-22]. In some individuals, behavioural indicators of pain temporally precede episodes of self-injury, suggesting that pain may cause, rather than result from, self-injury [23]. Given the association between pain and challenging behaviour, the increased likelihood of individuals with TSC experiencing pain suggests that this would be a high-risk group for self-injurious and aggressive behaviours.

Tuberous sclerosis complex is also associated with autism spectrum disorder (ASD). Estimated prevalence rates of TSC in people with ASD range from 1% up to 14% in individuals who also experience seizures and 40 to 45% of individuals with TSC may meet criteria for an ASD [24-27]. The most systematic evaluation of ASD in TSC was conducted by Bolton *et al*., [28]. Using the revised Autism Diagnostic Interview [29], the generic Autism Diagnostic Observation Schedule [30], developmental histories, cognitive test results and consensus diagnoses between two psychiatrists, 35.85% of individuals with TSC met ICD-10 (International Classification of Disease, 10th edition) [31] criteria for an ASD diagnosis. An ASD diagnosis and autism symptomatology are associated with increased risk of aggression and self-injury in people with ID, including those with genetic syndromes such as Down, fragile X, Prader-Willi and Cornelia de Lange syndromes [12,26,32-34].

Comparing the rates of behaviour between different syndrome groups has proven informative in previous studies [32]. Arron *et al*. [32] found that self-injury was significantly more prevalent in Cri du Chat, Cornelia de Lange, fragile X, Prader-Willi, Lowe and Smith-Magenis syndromes, and aggression was significantly higher in people with Angelman and Smith-Magenis syndromes relative to a contrast group. Furthermore, it was also shown that some topographies of self-injury were more prevalent in certain syndromes. It would be useful to use a methodology such as that described in the Arron *et al*. paper [32] to compare the rates of these behaviours in TSC with other genetic syndrome groups having well-documented behavioural phenotypes, in order to ascertain whether TSC is a high-risk group for self-injury and aggression.

In addition to the association of level of ID, pain and ASD symptomatology with self-injury and aggression, research into other person characteristics in different syndrome groups has identified additional behavioural correlates. These include hyperactivity [32,35-37], impulsivity [32,34-37], low mood [32,37,38] and repetitive behaviours [35-37]. Currently, it is not known whether these characteristics are associated with self-injury and aggression in children with TSC. Understanding the characteristics associated with these behaviours may inform causal models across different syndrome groups including TSC, which, in turn, could have implications for intervention.

Currently, the most well-established causal model of challenging behaviour is the operant learning theory [39]. This model states that behaviours are inadvertently reinforced by environmental consequences, such as the delivery of social attention or the removal of aversive tasks [40,41]. Through reinforcement, behaviours are shaped into challenging behaviours, such as self-injury and aggression. Although there is a wealth of evidence in support of the operant learning theory [41-43], it is not able to account for the differences in rates of challenging behaviour between different syndrome groups or explain why certain person characteristics are associated with challenging behaviour [32,35-39]. It is, therefore, important to gain information to inform alternative theories alongside the operant learning theory, to provide a more comprehensive account of challenging behaviour.

In summary, the behavioural and physical characteristics associated with TSC suggest that individuals with TSC might be at high risk of self-injury and aggression. However, no comparison including people with TSC has been conducted to test this hypothesis. Also, previous research has demonstrated an association between several person characteristics and self-injury and aggression. Again, these associations have yet to be assessed in individuals with TSC.

There are two aims to this study:

1. To compare the rates of self-injury and aggression and different topographies of self-injury in children with TSC with those with other genetic syndromes associated with ID.

2. To compare levels of negative affect, autism spectrum behaviours, hyperactivity, repetitive and impulsive behaviours and behavioural indicators of pain in children with TSC who engage in self-injury and aggression with children with TSC who do not engage in these behaviours.

## Methods

### Recruitment and participants

Participants with TSC were recruited as part of an ongoing survey at the Cerebra Centre for Neurodevelopmental Disorders [32,44,45]. Parents or carers of individuals with TSC under the age of 16 were invited to complete the questionnaires on behalf of their children or the children under their care, and acted as respondents. The data presented in this paper were collected for the purpose of an ongoing study at the Cerebra Centre for Neurodevelopmental Disorders. Potential participants were approached through various family syndrome support groups and invited to participate. Many of the support groups do not hold demographic information about their members so invitations were sent to their entire memberships. Therefore, it is not possible to estimate a response rate for this study.

Four comparison groups were selected because they were broadly similar to the TSC group in terms of ability level (measured by the Wessex Behaviour Schedule [46]). These were ASD, Cornelia de Lange, fragile X and Down syndromes. Data relating to these individuals were accessed from a database at the Cerebra Centre for Neurodevelopmental Disorders. Down syndrome acted as the main comparison group because it has a well-documented behavioural phenotype [47-50]. Prevalence rates of self-injury and aggression have been shown to be similar in individuals with Down syndrome and individuals with ID of heterogeneous aetiology, so Down syndrome will act as a homogenous, well-documented contrast group [48,49,51].

Children younger than four years were excluded, as one measure required informants to rate behaviour when the child was four to five years old. Therefore, participants were aged between 4 and 15 years 11 months.

Individuals within the ASD group were screened to ensure that they scored above the cut-off for ASD on the Social Communication Questionnaire. Individuals with TSC, Cornelia de Lange, fragile X and Down syndromes were included if their diagnosis had been confirmed by a paediatrician or clinical geneticist. Table 1 provides a description of the participants.

**Table 1:** **Demographic characteristics*; *age, sex, mobility, hearing and vision status, verbal ability and self-help skills**

	***N***	**Mean age, years (SD)**	**% male (*N*)**	**% verbal or partly verbal**^**a **^**(*N*)**	**% mobile (*N*)**	**% able or partly able**^**b **^**(*N*)**	**% normal vision (*N*)**	**% normal hearing (*N*)**
Down syndrome (comparison group)	43	9.00 (3.31)	41.9 (18)	95.2^c^ (40)	83.7 (36)	90.7 (39)	55.8 (24)	53.5 (23)
Tuberous sclerosis complex	37	10.08 (3.09)	51.4 (19)	89.2 (33)	81.1 (30)	78.4 (29)	**89.2** (33)	**97.2**^**c**^ (35)
Cornelia de Lange syndrome	61	10.10 (3.25)	44.3 (27)	**62.7**^**c**^ (37)	**50.0**^**c**^ (30)	**44.3** (27)	66.7^c^ (40)	57.4 (35)
fragile X syndrome	112	**10.88 (2.58)**	**100** (112)	95.5 (107)	69.4^c^ (77)	89.3 (100)	**87.5** (98)	**97.3** (108)
Autism spectrum disorder	188	9.37 (3.14)	**85.6** (161)	93.0^c^ (174)	**94.7**^**c**^ (177)	87.2 (164)	**96.8** (182)	**97.3** (183)

^a^Based on the speech item of the Wessex Behaviour Schedule. Verbal or partly verbal is defined as a score of ≥2. ^b^Based on the self-help scale of the Wessex Behaviour Schedule. Able or partly able is defined as a score of >2. ^c^Data missing from one participant. **Bold** indicates whether the value is significantly different compared with the Down syndrome comparison group (*P* < 0.05).

### Procedure

The Tuberous Sclerosis Association, a UK not-for-profit organization for users and carers, sent information sheets, consent forms and questionnaires to their membership.

### Measures

The following measures are all informant-based questionnaires. The revised Non-communicating Children Pain Checklist (NCCPC-R) was only completed by parents and carers of children with TSC.

#### Challenging Behaviour Questionnaire ( [52])

This is a brief questionnaire consisting of eight items. Four questions evaluate the presence of self-injury, physical aggression, destruction of property and stereotyped behaviours over the previous month. The remaining four items require the informant to state the topography of self-injury and provide details about the severity of self-injury, including how long the longest episode of behaviour lasted, whether physical contact or restraint was required and how frequently the behaviour occurred. The questionnaire has good interrater reliability with reliability coefficients ranging from 0.46 to 0.72 [52].

#### Mood, Interest and Pleasure Questionnaire, Short version (MIPQ [53])

This is used to examine affect in individuals with severe and profound ID and contains two subscales; the ‘mood’ subscale and the ‘interest and pleasure’ subscale, based on two main constructs of depression listed in the DSM-IV. Ratings are made following observations over a two-week period. Examination of the psychometric properties of the MIPQ demonstrated good test-retest and interrater reliability scores with *κ* values of 0.87 and 0.76, respectively [54]. Internal consistency was 0.94. Evidence to support construct validity was obtained by correlating scores with the ‘lethargy and social withdrawal’ scale on the Aberrant Behavior Checklist [55].

#### Wessex Behaviour Schedule [46]

The Wessex Behaviour Schedule is used to measure adaptive behaviour and provides a proxy measure of degree of ID. It comprises two subscales, the ‘social and physical incapacity’ (SPI) subscale and the ‘speech, self-help and literacy’ (SSL) subscale, although only the second subscale was used in this study. Interrater reliability of this measure has been reported as percentage agreement on responses. Reliability for the overall score on the SPI subscale is reported at 65%, reliability for the overall score on the SSL scales is reported at 76% [56].

#### The Activity Questionnaire (TAQ [57])

This measure is used to assess the presence of impulsive behaviours and overactivity in people with ID. This measure is suitable for verbal and non-verbal individuals. Internal consistency for both the full scale and subscales is good, with all subscales positively correlating with each other (*P* < 0.001); overactivity and impulsivity (*r*(755) = 0.59), overactivity and impulsive speech (r(517) = 0.50) and impulsivity and impulsive speech (*r*(517) = 0.50) [58]. Interrater and test-retest reliability are reported to be good. Correlations for all subscales are at 0.70 or above [59].

#### Social Communication Questionnaire (SCQ [60,61])

This measure is used to rate the presence of behaviours associated with ASD. Nineteen out of 40 items rate the child’s current behaviour and the remainder ask questions relating to behaviour when aged four to five years. A clinical cut-off of 15 or more on the SCQ is suggestive of ASD and a cut-off of 22 is suggestive of autism [58,60]. A score of 15 has a specificity of 0.80 and a sensitivity of 0.96 when differentiating individuals with pervasive developmental disorders from other diagnoses (not including people with ID) and a specificity of 0.67 and sensitivity of 0.96 when differentiating individuals with ASD from those with ID. A score of 22 is associated with a specificity of 0.60 and a sensitivity of 0.75 for differentiating individuals with autism from other pervasive developmental disorders [61].

#### The Repetitive Behaviour Questionnaire (RBQ [62])

The Repetitive Behaviour Questionnaire (RBQ) is an informant questionnaire for use in relation to children and adults with ID and is suitable for use with both verbal and non-verbal individuals. The RBQ is used to examine the presence and frequency of 19 different repetitive behaviours including stereotyped behaviours, compulsive behaviours, repetitive vocalizations, obsessions and insistence on sameness. This measure has good interrater and test-retest reliability, with Spearman coefficients ranging from 0.46 to 0.80 and 0.61 to 0.93, respectively [44]. The RBQ also has good concurrent and content validity (0.6, *P* < 0.001) when compared with the Autism Screening Questionnaire [60].

#### The revised Non-communicating Children’s Pain Checklist (NCCPC-R [63])

Informants using the NCCPC-R are required to rate the frequency of behaviours related to pain in children with ID. The NCCPC-R has good internal validity when used retrospectively [64,65]; there is high inter-episode consistency between two separate episodes of pain and consistent behaviour ratings when no pain is present [66]. For the purpose of this study, the administration of the NCCPC-R was modified. Respondents were asked to rate the frequency of behaviour over a week rather than over two hours. This modification was made to identify individuals likely to be experiencing chronic but potentially intermittent health conditions and pain as opposed to episodes of acute pain. This modification has been used previously to measure ‘typical’ pain behaviour [66,67].

### Data analysis

Data were analyzed using Statistical Package for Social Sciences (SPSS) 18.0 software. All data were tested for normality using Shapiro-Wilk tests and non-parametric tests were used where necessary. Odds ratios were used to indicate whether challenging behaviour was significantly more likely to occur in Down syndrome than in TSC and other genetic syndromes. The odds of something happening is defined as the probability of an event occurring divided by the probability of the event not occurring [68]. In this case, the odds ratio is the odds of the challenging behaviour occurring in the test syndrome group (that is, TSC) divided by the odds of challenging behaviour occurring in the comparison group (that is, Down syndrome). This is different from relative risk, which is a calculation that divides the risk of an event occurring in one situation by the risk of an event occurring in a separate situation (that is, the risk of challenging behaviour in TSC divided by the risk of challenging behaviour in Down syndrome). The odds ratio was deemed significant if the lower bound of the confidence interval was greater than one. When using 95% or 99% confidence intervals, confidence intervals that exceed one would represent a difference in odds at a significance level of *P* < 0.05 and *P <* 0.01, respectively. Down syndrome was selected as the primary contrast group, with which other groups, including TSC were compared. Down syndrome represents a homogenous group with a well-documented behavioural phenotype, meaning that comparisons between Down syndrome and other syndrome groups, including TSC, are informative within the ID literature. Mann-Whitney *U* tests were conducted to compare person characteristics between individuals with and without self-injury and aggression in children with TSC. Effect size was calculated manually for the Mann-Whitney *U* tests by dividing *Z* by the square root of *N*.

## Results

### Cross-syndromal comparison of rates of self-injury and aggression and topographies of self-injury

Odds ratios were used to compare parental reports of rates of self-injury and aggression between individuals with TSC and Down syndrome. The odds ratios of self-injury and aggression were also compared in individuals with fragile X and Cornelia de Lange syndromes and ASD with individuals with Down syndrome, to contrast the risk of given behaviours across syndrome groups and provide further reference points for the group with TSC. Odds ratios were also used to compare the risk of different topographies of self-injury in individuals with TSC to individuals with Down syndrome. Again, the odds ratios of topographies of self-injury in people with ASD, fragile X, Cornelia de Lange syndromes compared with Down syndrome were also reported, to provide further contrasts.

The results shown in Table 2 indicate that reported rates of self-injury and aggression in TSC were high (self-injury, 27%; aggression, 50%), but the risk of these behaviours occurring in children with TSC was not significantly different from the risk of the behaviours occurring in children with Down syndrome. As expected, the risk of self-injury was significantly greater in people with Cornelia de Lange syndrome and fragile X syndrome than in people with Down syndrome and the risk of self-injury and aggression was significantly greater in people with ASD than in people with Down syndrome.

**Table 2:** Rates of self-injury and aggression compared between Down syndrome and other syndromes, including TSC

	**Self-injurious behaviour**		**Aggression**	
**Syndrome group**	**%**	**Odds ratio between syndrome groups (99% confidence intervals)**	**%**	**Odds ratio between syndrome groups (99% confidence intervals)**
Down syndrome (comparison group)	11.90^a^	–	40.50^a^	–
Tuberous sclerosis complex	27.00	2.74 (0.58 to 13.00)	50.00^a^	1.47 (0.45 to 4.80)
Cornelia de Lange syndrome	63.90	**13.12 (3.21 to 53.66)**	44.10^b^	1.16 (0.40 to 3.33)
Fragile X syndrome	54.50	**8.85 (2.36 to 33.24)**	60.90^b^	2.29 (0.88 to 5.95)
Autism spectrum disorder	41.70^a^	**5.30 (1.46 to 19.19)**	66.70^c^	**2.94 (1.19 to 7.29)**

^a^Data missing for one participant; ^b^Data missing for two participants; ^c^Data missing for eight participants; **Bold** text indicates significantly greater risk of challenging behaviour in test group compared with Down syndrome comparison group (*P* < 0.01).

There was no significant difference in the risk of any topography of self-injury in people with TSC compared with people with Down syndrome. This finding was also replicated across the other syndrome groups tested.

### Differences in person characteristics between children with and without self-injury and aggression in TSC

The second aim of this study was to investigate whether characteristics previously reported to be associated with self-injury and aggression in other syndrome groups were also associated with these behaviours in children with TSC. Student’s *t* tests were conducted to assess the difference in age between people with and without challenging behaviour in individuals with TSC. Chi square analysis was used to determine whether the proportion of individuals engaging in challenging behaviour was different between people with hearing and vision problems and those without; between individuals with good and poor mobility and self-help skills and between individuals with and without the ability to speak full words. In addition to demographic variables, other person characteristics were compared between children with and without self-injury and aggression in the TSC group. These included level of affect, socialization and communication difficulties, hyperactivity, impulsivity, compulsive, stereotyped and repetitive behaviours, and behavioural indicators of pain. Mann-Whitney *U* tests (and independent sample *t* tests where appropriate) were conducted to compare scores between individuals with and without self-injury and aggression in children with TSC. Tables 3 and 4 show the results of these analyses.

**Table 3:** Comparison of demographic characteristics between individuals with TSC with and without self-injury and aggression

	**Self-injury**				**Aggression**			
	**Present**	**Absent**	***t *or *χ***^***2***^	***P***	**Present**	**Absent**	***t *or *χ***^***2***^	***P***
Age^a^ (mean)	8.00	10.85	2.70	**0.01**	9.17	10.89	1.70	0.10
Sex (% male)	30.00	59.26	2.50	0.15	38.89	61.11	1.78	0.32
Self-help (% partly able or able^d^)	50.00	88.89	6.51	**0.02**	72.22	83.33	0.64	0.69
Mobility^c^ (% fully mobile)	80.00	81.48	0.01	1.00	83.33	77.78	0.18	1.0
Vision^c^ (% normal)	80.00	92.59	1.20	0.29	88.89	94.44	0.36	1.0
Hearing^c^ (% normal)	100.00	96.15	0.40	1.00	100.00	94.12	1.09	0.49
Speech^c^ (% partly verbal or verbal^d^)	80.00	92.59	1.20	0.29	94.44	83.33	1.13	0.60

^a^Age in years. ^b^Based on the self-help scale of the Wessex Behaviour Schedule. Able or partly able is defined as a score of >2. ^c^Taken from the Wessex Behaviour Schedule. ^d^Based on the speech item of the Wessex Behaviour Schedule. Verbal or partly verbal is defined as a score of ≥2. **Bold** text indicates whether there is a significant difference in demographic variables between individuals with and without challenging behaviour.

**Table 4:** Comparison of person characteristics between individuals with TSC with and without self-injury and aggression

**Measure**	**Subscale or person characteristic assessed**	**Median (interquartile range) self-injury**				**Median (interquartile range) aggression**			
		**Present**	**Absent**	***U *score**	**Effect size**	**Present**	**Absent**	***U *score**	**Effect size**
MIPQ	Mood	16.00 (13.00 to 17.25)	21.00 (18.00 to 24.00)	32.00***	−0.59 (large)	17.50 (15.00 to 21.00)	22.00 (17.50 to 24.00)	79.50**	−0.43 (medium-large)
	Interest and pleasure	15.00 (8.00-17.50)	19.00 (13.50 to 21.25)	70.50*	−0.37 (medium)	17.50 (11.75 to 20.00)	19.00 (12.00 to 21.50)	140.00	−0.12 (small)
SCQ	Communication	9.00 (5.88 to 10.75)	5.00 (4.00 to 9.00)	92.50	−0.24 (small to medium)	6.75 (4.00 to 9.00)	5.00 (3.50 to 10.69)	158.50	−0.02 (small)
	Socialization	8.50 (4.50 to 13.00)	5.00 (2.00 to 10.00)	92.00	−0.25 (small to medium)	6.50 (4.75 to 10.00)	3.00 (2.00 to 11.50)	132.50	−0.16 (small)
	Repetitive behaviour	4.50 (3.25 to 6.25)	3.50 (1.00 to 6.00)	99.50	−0.21 (small to medium)	4.50 (3.25 to 6.25)	3.00 (1.00 to 4.50)	97.50*	−0.16 (small)
TAQ	Overactivity	21.00 (16.50 to 29.75)	5.50 (2.00 to 16.00)	62.00*	−0.42 (medium to large)	19.50 (8.00 to 28.25)	5.00 (1.00 to 7.00)	67.50**	−0.38 (medium)
	Impulsivity	22.50 (15.75 to 24.00)	8.50 (2.75 to 19.00)	50.00**	−0.48 (medium to large)	19.50 (10.00 to 23.00)	6.00 (1.00 to 16.50)	70.00*	−0.46 (medium to large)
RBQ	Compulsive behaviour	0.00 (0.00 to 17.50)	2.00 (0.00 to 5.25)	117.00	−0.11 (small)	2.00 (0.00 to 15.50)	1.00 (0.00 to 3.50)	132.00	−0.16 (small)
	Stereotyped behaviour	9.00 (2.75 to 12.00)	0.00 (0.00 to 4.25)	55.50*	−0.47 (medium to large)	5.50 (0.00 to 9.25)	0.00 (0.00 to 2.00)	91.00*	−0.39 (medium)
	Insistence on sameness	3.50 (2.75 to 5.50)	2.00 (0.00 to 4.00)	77.50*	−0.34 (medium)	3.00 (1.50 to 5.50)	1.50 (0.00 to 3.25)	104.00	−0.31 (medium)
	Repetitive use of language*	9.00 (5.00 to 11.00)	3.50 (0.00 to 5.75)	30.50*	−0.46 (medium to large)	7.00 (2.75 to 10.00)	2.000 (0.00 to 5.00)	54.50*	−0.47 (medium to large)
	Restricted preferences*	6.00 (4.00 to 8.00)	4.00 (0.00 to 7.00)	57.00	−0.23 (small to medium)	5.00 (4.00 to 7.75)	1.00 (0.00 to 5.00)	74.50	−0.33 (medium)
NCCPC-R	Pain indicators	26.00 (18.50 to 39.63)	9.00 (4.75 to 14.75)	27.00***	−0.62 (large)	20.00 (13.00 to 35.00)	7.00 (3.00 to 10.00)	39.00***	−0.65 (large)

*P < 0.05, **P < 0.01, ***P < 0.001. MIPQ, Mood, Interest and Pleasure Questionnaire; NCCPC-R, Non-communicating Children Pain Checklist; RBQ, Repetitive Behaviour Questionnaire; SCQ, Social Communication Questionnaire; TAQ, the Activity Questionnaire.

Table 3 shows that individuals who engaged in self-injury were significantly younger and reported to have lower levels of adaptive behaviour (that is, they were less able to wash, dress and feed themselves independently) than those who did not self-injure. No other significant differences were found in demographic variables between individuals with and without self-injury or aggression. Results presented in Table 4 show that parental reports of affect and levels of interest and pleasure were significantly lower in children reported to engage in self-injury than in those who were not. Parents’ ratings of activity, impulsivity, insistence on sameness, repetitive language, stereotyped behaviours and behavioural indicators of pain were significantly higher in individuals reported to engage in self-injury than in those who did not show this behaviour. The results in Table 4 also show that individuals reported to engage in aggressive behaviour had significantly lower affect and higher levels of activity, repetitive language and repetitive, impulsive, stereotyped behaviours and a higher number of behavioural indicators of pain compared to individuals who did not engage in aggression. Each significant difference was associated with a medium-large effect size.

### Assessing the potentially confounding influence of level of intellectual disability and age

As shown in Table 3, children with TSC who were reported to engage in self-injurious behaviour were significantly younger and had lower levels of adaptive behaviour than those who did not engage in self-injurious behaviour. To evaluate whether these differences confounded the association between person characteristics (such as mood, levels of activity and behavioural indicators of pain) and self-injury, a binary logistic regression analysis was conducted with self-injury (present or absent) as the dependent variable. The predictor variables of interest were those shown to differ significantly between individuals who did and did not engage in self-injury. The predictive value of these variables was assessed after factoring out the influence of age and level of adaptive functioning. Table 5 shows the results of this analysis. When controlling for age, all of the variables shown to be significantly association with self-injury (mood, interest and pleasure, impulsive behaviour, overactivity, stereotyped behaviour, insistence on sameness and pain) remained so once the influence of age was removed. When controlling for level of adaptive behaviour, interest and pleasure, insistence on sameness and impulsivity were no longer significantly associated with the presence of self-injury.

**Table 5:** Binary logistic regression; the association between person characteristics and self-injury, controlling for ID and age

	***Β***	***SE *β**	**Wald’s *χ***^***2***^	***P***	***E***^**β **^**(odds ratio)**
**Controlling for level of intellectual disability**					
Self-injury present × MIPQ; mood subscale	−0.51	0.21	5.99	**0.01**	0.60
Self-injury present × MIPQ; interest and pleasure subscale score	−0.12	0.09	1.82	0.18	0.89
Self-injury present × TAQ; impulsivity subscale	0.15	0.07	5.30	**0.02**	1.16
Self-injury present × TAQ; overactivity subscale	0.08	0.04	3.83	0.05	1.08
Self-injury present × RBQ; stereotyped behaviour subscale	0.22	0.10	4.65	**0.03**	1.25
Self-injury present × RBQ; insistence on sameness	0.31	0.18	3.08	0.08	1.36
Self-injury present × RBQ; repetitive use of language^a^	0.34	0.16	4.75	**0.03**	1.40
Self-injury present × NCCPC total	0.13	0.06	5.02	**0.03**	1.14
**Controlling for age**					
Self-injury present × MIPQ; mood subscale	−0.70	0.29	5.84	**0.02**	0.50
Self-injury present × MIPQ; interest and pleasure subscale score	−0.25	0.10	6.00	**0.01**	0.78
Self-injury present × TAQ; impulsivity subscale	0.15	0.07	4.84	**0.03**	1.17
Self-injury present × TAQ; overactivity subscale	0.08	0.04	4.14	**0.04**	1.08
Self-injury present × RBQ; stereotyped behaviour subscale	0.24	0.10	5.38	**0.02**	1.27
Self-injury present × RBQ; insistence on sameness	0.58	0.25	5.21	**0.02**	1.78
Self-injury present × RBQ; repetitive use of language^a^	0.34	0.16	4.88	**0.03**	1.41
Self-injury present × NCCPC total	0.12	0.05	7.07	**0.01**	1.13

^a^Verbal participants only; **Bold** text indicates significant associations between self-injury and person characteristics that remain significant after the influence of level of intellectual disability and age are controlled for.

## Discussion

Parental reports of rates of self-injury and aggression in children with TSC were 27% and 50%, respectively. Although not statistically significant, the reported rate of self-injury in TSC was more than twice as high as in Down syndrome and the reported rate of aggression was also higher. These results are consistent with previous research in individuals with TSC, although the rate of self-injurious behaviour reported in this study was at the higher end of estimates [9-11]. These results suggest that individuals with TSC are at a high risk of self-injury and aggression, although they are not significantly more likely to engage in these behaviours than individuals from other genetic syndrome groups with similar levels of ID. This finding is surprising, given the significant proportion of individuals with TSC with profound ID, the high levels of ASD and ASD symptomatology found in TSC and the high risk of pain and discomfort associated with the syndrome, which are all risk markers for self-injury and aggression [12].

There are a number of explanations for this finding. Firstly, there was a relatively small sample of children with TSC in this study (*N* = 37). This may have resulted in there being insufficient statistical power to identify a significant difference between the rates of challenging behaviour in TSC and other genetic syndrome groups. Alternatively, it could be the case that the children with TSC included in this study had a broader range of ID than expected. The Wessex Behaviour Schedule, in which informants are asked to rate their child’s ability to complete self-help tasks independently, was the only proxy measure of ID used in this study. During early childhood years, a child would not typically be able to perform these tasks without help but according to the Wessex Behaviour Schedule they would still fall under the category ‘not able’. Therefore, although the TSC group appeared to be well-matched with the comparison groups in terms of intellectual ability, it might be the case that a greater proportion of the younger children in the TSC had no ID or mild ID compared with the other syndrome groups, which would make them less likely to engage in self-injury and aggression. It would be useful for future research to compare rates of behaviour in TSC with other syndrome groups when level of ID is assessed using direct cognitive assessments. Rates of self-injury and aggression in individuals with TSC would be expected to be higher than those reported in this study if only children with ID were included. Significant differences between rates of self-injury and aggressive behaviour in children with TSC compared with other syndrome groups may then be observed.

The second finding of this study is that person characteristics were associated with increased risk of self-injury and aggression in children with TSC. Self-injurious behaviour was more likely to occur in individuals with TSC who were reported to have lower levels of adaptive functioning and who were younger. Negative affect, higher levels of impulsivity and activity, ASD symptomatology, including stereotyped behaviour, insistence on sameness and repetitive use of language, and behavioural indicators of pain were all associated with self-injury. The results were broadly similar when assessing person characteristics associated with aggression. Children who engaged in aggressive behaviour had lower moods and higher levels of activity, impulsivity, repetitive behaviour, stereotyped behaviour and repetitive use of language than children who did not engage in aggressive behaviour. Again, a higher number of behavioural indicators of pain were observed in children with aggression than in those without. These results are in line with previous research findings with other syndrome groups [32]. There may be value in using these person characteristics or indicators of pain and discomfort to identify individuals with ID, including those with TSC, who are more likely to engage in challenging behaviour.

Differences in rates of challenging behaviour between and within syndrome groups might demonstrate a greater influence of internal factors on challenging behaviour, as opposed to environmental effects. This suggests that, in some cases at least, internal factors, such as pain and discomfort might contribute towards the development of self-injurious and aggressive behaviours. Currently, the dominant theory for explaining how and why challenging behaviour develops is the operant learning theory [39,69]. As stated previously, this model states that behaviours are inadvertently reinforced by environmental consequences, such as the delivery of social attention or the removal of aversive tasks [40,41]. Through reinforcement, behaviours are shaped into challenging behaviours, such as self-injury and aggression. If operant learning processes were the only explanation for challenging behaviour, rates of challenging behaviour would be expected to be constant across all syndrome groups and irrespective of person characteristics. Given that this is not the case, the findings of this study testify to the importance of understanding the role that other factors play in influencing challenging behaviour. Combining this knowledge with the already well-established literature regarding operant learning principles [41-43] could influence how challenging behaviours are understood and treated in people with ID [70,71].

Further research is required to better understand the influence of pain on challenging behaviour in individuals with TSC. The association between pain and aggressive behaviour could be explained by pain acting as a setting event in an operant conceptualization [19,69,72]. This means that the presence of pain increases the likelihood of self-injury and aggression during situations that are typically associated with these behaviours, such as during times of high cognitive demand [73]. In regards to self-injury, previous literature suggests that self-injury could moderate the perception of pain caused by ongoing health problems [74,75] and thus, underlying pain causes self-injury. Therefore, the presence of self-injury or aggressive behaviour in people with TSC could indicate the possibility of an underlying health condition. This could have huge clinical implications for identifying and treating health conditions and pain in people with TSC, given the complex health problems found in this group [13].

Another finding of this study was that the level of adaptive functioning was associated with self-injury, which is similar to previous findings that reported self-injury prevalence rates of 69%, 34% and 17% in people with severe or profound ID, mild ID and no ID, respectively [76]. When level of ability was controlled for, significant associations between self-injury and activity level, and between self-injury and levels of interest and pleasure, were no longer significant. It might be suggested that individuals with lower levels of adaptive functioning also have more clinical features of TSC, and are therefore more likely to suffer pain linked to health problems [10]. Thus, by controlling for ability, important differences in health and pain could also be lost, which could explain the findings reported in this study, as hyperactivity and low levels of interest and pleasure might be indirect indicators of pain [63,77,78]. This issue would also emerge from research with other genetic syndromes where multiple systems within the body are affected, such as Cornelia de Lange syndrome [79], Williams syndrome [80] and CHARGE syndrome [81]. Therefore, it is important at this stage not to regard with certainty apparent associations between person characteristics and challenging behaviour as being an artefact of increased levels of ID.

## Conclusions

The results from this study show that individuals with TSC are at a high risk of engaging in self-injury and aggression and are more likely to show these behaviours if they have particular person characteristics, including low mood, high levels of activity and ASD symptomatology. It was also shown that children with TSC who engage in self-injury and aggression are more likely to display behavioural indicators of pain. These findings testify to the importance of investigating the role of internal influences on self-injury and aggression in people with ID.

## Abbreviations

ASD: autism spectrum disorder; ICD-10: International Classification of Disease, 10^th^ edition; ID: intellectual disability; MIPQ: Mood, Interest and Pleasure Questionnaire; NCCPC-R: Non-communicating Children Pain Checklist; RBQ: Repetitive Behaviour Questionnaire; SCQ: Social Communication Questionnaire; SPI: social and physical incapacity; SPSS: Statistical Package for Social Sciences; SSL: speech, self-help and literacy; TAQ: the Activity Questionnaire; TSC: tuberous sclerosis complex.

## Competing interests

The authors declare that they have no competing interests.

## Authors’ contributions

KEE contributed to the design of the study, collected and analyzed the data and drafted the manuscript. CO, PJdV and JM contributed to the design of the study and were involved in revising the manuscript. JM and CR were involved in the data collection for the comparison groups. All authors read and approved the final version of the manuscript.
